# Nomogram based on dual-energy CT-derived extracellular volume fraction for the prediction of microsatellite instability status in gastric cancer

**DOI:** 10.3389/fonc.2024.1370031

**Published:** 2024-05-24

**Authors:** Wenjun Hu, Ying Zhao, Hongying Ji, Anliang Chen, Qihao Xu, Yijun Liu, Ziming Zhang, Ailian Liu

**Affiliations:** ^1^ Department of Radiology, First Affiliated Hospital of Dalian Medical University, Dalian, Liaoning, China; ^2^ Dalian Engineering Research Center for Artificial Intelligence in Medical Imaging, Dalian, Liaoning, China; ^3^ Department of Pathology, The First Affiliated Hospital, Dalian Medical University, Dalian, Liaoning, China; ^4^ College of Medical Imaging, Dalian Medical University, Dalian, Liaoning, China

**Keywords:** dual-energy CT, extracellular volume fraction, gastric cancer, microsatellite instability, nomogram

## Abstract

**Purpose:**

To develop and validate a nomogram based on extracellular volume (ECV) fraction derived from dual-energy CT (DECT) for preoperatively predicting microsatellite instability (MSI) status in gastric cancer (GC).

**Materials and methods:**

A total of 123 patients with GCs who underwent contrast-enhanced abdominal DECT scans were retrospectively enrolled. Patients were divided into MSI (n=41) and microsatellite stability (MSS, n=82) groups according to postoperative immunohistochemistry staining, then randomly assigned to the training (n=86) and validation cohorts (n=37). We extracted clinicopathological characteristics, CT imaging features, iodine concentrations (ICs), and normalized IC values against the aorta (nICs) in three enhanced phases. The ECV fraction derived from the iodine density map at the equilibrium phase was calculated. Univariate and multivariable logistic regression analyses were used to identify independent risk predictors for MSI status. Then, a nomogram was established, and its performance was evaluated by ROC analysis and Delong test. Its calibration performance and clinical utility were assessed by calibration curve and decision curve analysis, respectively.

**Results:**

The ECV fraction, tumor location, and Borrmann type were independent predictors of MSI status (all *P* < 0.05) and were used to establish the nomogram. The nomogram yielded higher AUCs of 0.826 (0.729–0.899) and 0.833 (0.675–0.935) in training and validation cohorts than single variables (*P*<0.05), with good calibration and clinical utility.

**Conclusions:**

The nomogram based on DECT-derived ECV fraction has the potential as a noninvasive biomarker to predict MSI status in GC patients.

## Introduction

Gastric cancer (GC) is one of the most aggressive malignancies, and its incidence and mortality rate are ranked fifth and fourth, respectively, among all cancers (1). However, the therapeutic response to primary treatment methods such as surgery, chemotherapy, and targeted therapy varies greatly among individuals, and the 5-year survival rate in patients with GC is less than 30% (2).

Microsatellite instability (MSI) is one of the molecular subtypes of GC. MSI status plays an important role in risk stratification and personalized treatment of GC patients, as MSI GC tissues have peculiar biological behaviors, including having a better prognosis, not benefiting from chemotherapy, and being sensitive to immune checkpoint inhibitor administration (3). In 2017, the US Food and Drug Administration announced that the monoclonal antibody drug pembrolizumab could be used to treat patients with MSI non-resectable or solid metastatic tumors (4). Thus, the 2021 National Comprehensive Cancer Network guidelines recommend routine testing of MSI status in all newly diagnosed GC patients (5). Currently, the primary methods to evaluate MSI status are immunohistochemistry (IHC) and polymerase chain reaction (PCR) (6), both of which rely on tissue samples obtained from gastroscopic biopsy or postoperative pathology. However, the invasive operation, spatial heterogeneity of MSI expression, complex procedures, and high cost have curtailed the use of these methods in clinical practice. Therefore, finding a noninvasive, cost-effective preoperative method to predict MSI status in GC is crucial.

Thus, many researchers have attempted to predict MSI status in GC using noninvasive imaging biomarkers. Prior research (7–9) has suggested that CT features and standardized uptake values in GC may be associated with MSI, but their predictive efficiencies and generalization capabilities are still unclear. In addition, many studies have shown that CT-based radiomics and deep learning algorithms (5, 10–12) have considerable potential for evaluating MSI status. However, the complex processes and low repeatability limit its clinical utility.

Dual-energy CT (DECT) is an advanced imaging technique that provides many quantitative parameters and qualitative features using two different energy CT data sets (13). The extracellular volume (ECV) fraction, which is calculated based on DECT, can achieve a noninvasive quantitative assessment of extracellular space and provide more information than conventional spectral parameters (14). It has been well established that the ECV fraction is useful for evaluating fibrosis in liver and heart (15, 16). Recently, investigators have attempted to study the potential value of ECV fraction in oncological assessments, such as the prediction of patient survival in unresectable pancreatic adenocarcinoma, grading of renal cell carcinoma, and prediction of treatment response in rectal cancer (17–19). However, there have not yet been any reports on testing the feasibility of ECV in the assessment of MSI status in GC.

The study aims to develop and validate a nomogram based on DECT-derived ECV fraction for preoperative prediction of MSI in GC, which may provide new help for the individualized treatment options and prognostic assessment of GC.

## Materials and methods

### Patients

**Figure 1:**
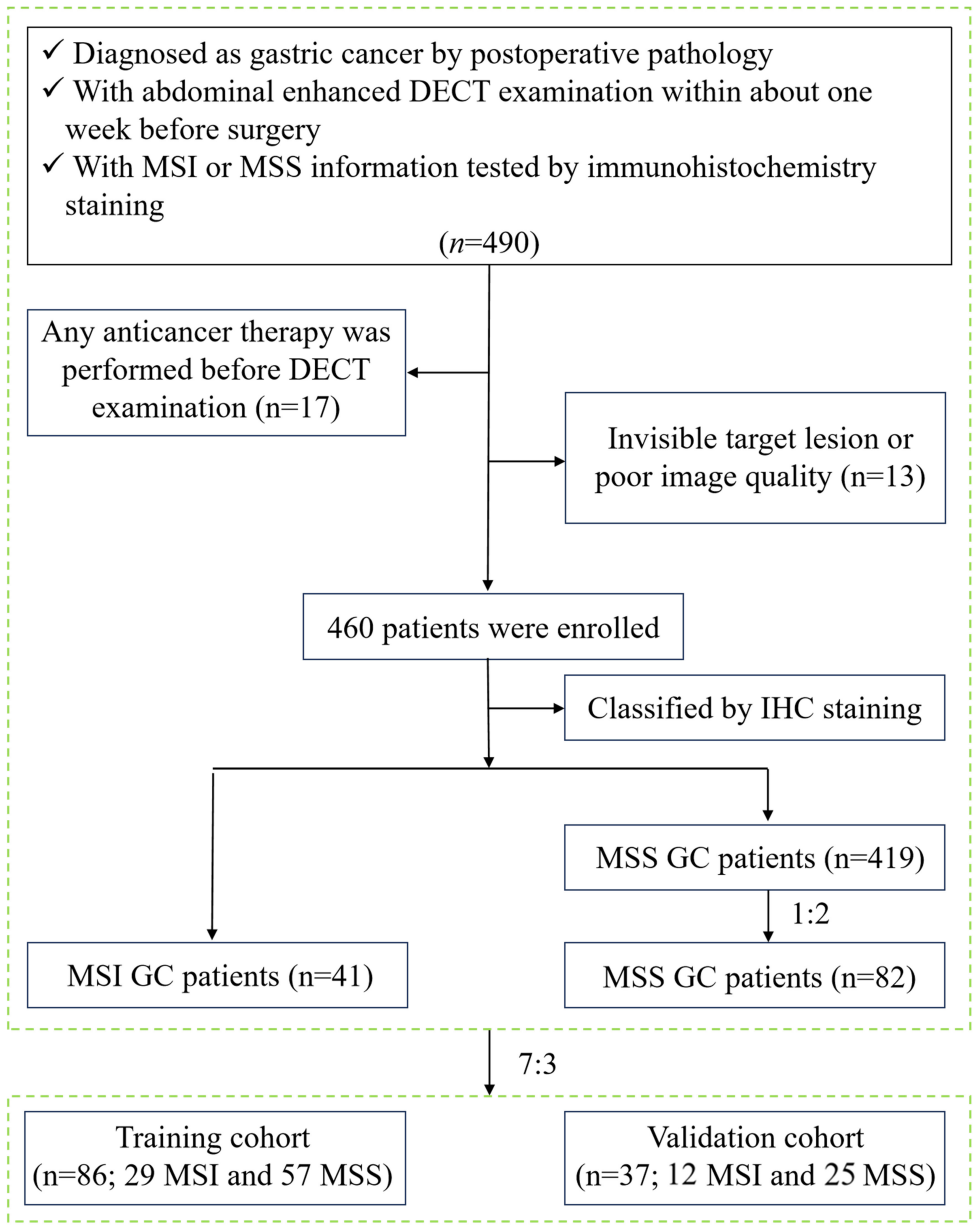
Flow chart of case enrollment. √ meets this inclusion criterion.

This study was approved by the Medical Ethics Committee of our hospital (approval number: PJ-KS-KY-2022–421) and waived the requirement for informed consent. Patients diagnosed with GC from August 2017 to December 2021 were retrospectively analyzed. Inclusion criteria were: ① GC patients were pathologically confirmed by radical gastrectomy; ② all patients performed abdominal enhanced DECT examination within one week before the surgery; ③ MSI status was tested by postoperative immunohistochemistry staining. Exclusion criteria were: ① patients who received GC-related treatment before DECT examination, such as radiotherapy, chemotherapy, biotherapy, etc. (n=17); ② invisible target lesions on CT images or poor image quality caused by motion artifacts and unsatisfactory gastric distention (n=13). A total of 460 GC patients were collected based on the results of MSI testing in the pathological report, including 41 MSI GC patients and 419 MSS GC patients. To avoid diagnostic bias due to sample imbalance, 82 patients with MSS GC were ultimately included in the control group by random sampling in the ratio of MSI: MSS = 1:2. Then, all patients were divided into a training cohort (n = 86, 57 MSS and 29 MSI) and a validation cohort (n = 37, 25 MSS and 12 MSI) in a ratio of 7:3. The study flow chart is shown in **Figure 1**.

The demographic, pathological, and laboratory information of patients were recorded. The demographic data included age, gender, alcohol history, smoking history, and family history of cancer. The pathological data included tumor diameter, histological type, differentiation degree, tumor-lymph node-metastasis (TNM) stage, perineural invasion, and lymphovascular invasion. The laboratory variables, including hematocrit level, carcinoembryonic antigen (CEA), alpha-fetoprotein (AFP), and carbohydrate antigen (CA) 19–9, were measured within one week of CT.

### MSI status assessment

The MSI status was identified using the immunohistochemical staining of four mismatch repair gene protein products, including MLH1, MSH2, MSH6, and PMS2. Positive staining of all four proteins indicates MSS, whereas any protein with negative staining indicates MSI. Two expert pathologists who were unaware of patients’ clinicopathologic data analyzed the results. If the conclusions were inconsistent, they reached an agreement through consultation.

### CT protocol

The abdominal DECT scans were performed on a Revolution CT scanner (GE Healthcare, Milwaukee, WI, USA) with gemstone spectral imaging (GSI) mode. All patients were informed to fast for 6–12 h and drank 800–1000 ml warm water to distend the stomach 20 min before CT examination. The patients were also trained to use non-abdominal breathing and breath-holding. After a non-enhanced CT scan was performed, 300–500mgI/kg of nonionic contrast material (350mgI/mL iohexol [Omnipaque, GE Healthcare]) was administered via median cubital vein at a rate of 3–5ml/s, followed by flushing of 20ml saline at the same injection rate. The arterial phase (AP) scans were obtained at 20 s after attaining 100-HU density elevation in the descending aorta using a bolus-tracking technique. The portal venous (VP) phase scans were obtained at 28s after the arterial phase scanning. The equilibrium phase (EP) scans were acquired at 90s after the portal venous phase scanning ended. The imaging acquisition parameters were as follows: rapid switching between tube voltages of 80 kVp and 140 kVp; tube current, 375 mA; helical pitch, 0.992:1; rotation time, 0.6 s/rot; detector width, 80 mm; slice thickness/gap, 5/5 mm.

### Image interpretation

The CT images were transferred to an advanced workstation 4.7 (GE Healthcare, USA) for reconstructing iodine-based material decomposition (MD) images at 1.25-mm image slice thickness and interval using GSI Viewer software. Image analysis and data measurement were performed by two radiologists with 20 years (observer 1 [A.L.L.]) and 3 years (observer 2 [W.J.H.]) experience in abdominal CT interpretation who were blinded to the grouping.

First, the following CT features of GC were observed and recorded: ① tumor thickness was defined as the thickest diameter of the gastric tumor on the maximal cross-section; ② tumor location was subclassified as gastric cardia/fundus, gastric body, and gastric antrum; ③ Borrmann type:Borrmann type I was defined as a nodular polypoid mass, type II as an ulcerative mass with distinct borders, type III as an infiltrating ulcerating mass, and type IV as a diffuse thickening of the gastric wall (20); ④ serous invasion was defined as a nodular or irregular surface of the gastric wall or perigastric fat infiltration (21); ⑤ lymph node metastasis was defined as the short-axis diameter of an enlarged lymph node>10 mm or a cluster of ≥3 lymph nodes (22). All features were assessed based on the agreement between the two radiologists and final consensuses were obtained by discussion in case of disagreement.

Then, the two radiologists together selected the slice with the maximum cross-sectional tumor area and independently placed three circular regions of interest (ROIs) on the solid areas of the tumor in iodine-based MD images. The ROIs were at least 2 mm from the tumor margin and had no less than 50 mm^2^ areas. The ROIs included as much of the tumor region as possible while avoiding vessels, necrosis, and calcification portions. We adjusted the ROIs on images of different phases to ensure they were in the same location and size. The iodine concentrations (ICs) in the arterial phase (ICAP), venous phase (ICVP) and equilibrium phase (ICEP) were automatically generated. The averaged values of three measurements were calculated as the final results. At the same time, circular ROIs of about 100 mm^2^ were placed on the abdominal aorta at the same slices as the lesions measured above to obtain aortic ICs. Normalized ICs (nICs) were acquired by dividing tumor ICs with aortic ICs. The tumor DECT-derived ECV fraction was calculated as follows (14): ECV fraction (%) = (1-hematocrit) × (IClesion/ICaorta) ×100, where IClesion and ICaorta are iodine concentrations in the equilibrium phase of the tumor and aorta, respectively.

### Statistical analysis

Statistical analysis was performed using SPSS software (version 26.0), R software (version 4.1.0), and MedCalc software (version 19.1.2). The interclass correlation coefficient (ICC) was used to test the consistency of the measurement results between the two observers. The Shapiro-Wilk tests were used to test the normality assumption. The quantitative data were compared using the independent *t* test or Mann-Whitney *U* test. Categorical data were compared using the chi-square test or Fisher exact test. We entered variables in the training cohort for which *P*<0.05 was determined by the univariate analysis into a multivariate logistic regression to determine independent predictors of MSI status. Based on the relevant factors and regression coefficients obtained by multivariate logistic regression analysis, we developed a prediction model and visualized it as an easy-to-use nomogram. Internal validation of the nomogram was conducted using the fivefold cross-validation method. The validation cohort was used to evaluate the generalization capabilities of the nomogram. The receiver operating characteristic (ROC) curve was used to evaluate the predictive efficacy, and the area under the ROC curve (AUC), threshold, sensitivity, and specificity were obtained. The Delong test was used to compare the differences in the predictive efficacy. A *P* value <0.05 indicated statistical significance. Decision curve analysis (DCA) was conducted to assess the clinical practicability of the nomogram. The calibration curve and Hosmer-Lemeshow test were used to assess the nomogram calibration, and *P* > 0.05 indicated that the goodness-of-fit of the model is good.

## Results

### General data

A total of 123 patients were included in the study (83 males and 40 females; median age: 64 years, range: 36−82 years). There were 86 and 37 patients that finally enrolled in the training (61 males and 25 females; median age: 63.5 years, range: 35−81 years) and validation cohorts (22 males and 15 females; median age: 67 years, range: 32−82 years), respectively. There was no significant difference in the prevalence of MSI status (33.7% in the training cohort vs. 32.4% in the validation cohort; χ^2 =^ 0.019, *P*=0.889) and other clinicopathological features between the two cohorts. The tumor diameter was significantly higher in the MSI group than in the MSS group for the training cohort but not for the validation cohort. The incidences of earlier TNM stage and negative perineural invasion in the MSI group were higher than those in the MSS group in both cohorts (all *P* < 0.05) (**Table 1**).

**Table 1:** Clinicopathological characteristics in the training and validation cohorts.

Characteristics		Training cohort (*n*=86)			Validation cohort (*n*=37)			*P* ^†^
		MSS (n=57)	MSI (n=29)	*P^#^*	MSS (n=25)	MSI (n=12)	*P^#^*	
Age (years)		63.00 (58.00, 69.00)	64.00 (57.70, 69.30)	0.844	66.00 (57.70, 70.00)	68.50 (65.00, 76.75)	0.074	0.132
Sex	Male	42 (73.68%)	19 (65.52%)	0.430	15 (60.00%)	7 (58.33%)	1.000	0.213
	Female	15 (26.32%)	10 (34.48%)		10 (40.00%)	5 (41.67%)		
Alcohol history	No	48 (84.21%)	22 (75.86%)	0.347	22 (88.00%)	11 (91.67%)	1.000	0.283
	Yes	9 (15.79%)	7 (24.14%)		3 (12.00%)	1 (8.33%)		
Smoking history	No	41 (71.93%)	22 (75.86%)	0.697	20 (80.00%)	11 (91.67%)	0.641	0.207
	Yes	16 (28.07%)	7 (24.14%)		5 (20.00%)	1 (8.33%)		
Family history of cancer	No	51 (89.47%)	29 (100.00%)	0.173	24 (96.00%)	12 (100.00%)	1.000	0.607
	Yes	6 (10.53%)	0 (0.00%)		1 (4.00%)	0 (0.00%)		
CEA (ng/mL)		2.35 (1.37, 4.58)	1.70 (1.25, 2.88)	0.112	2.11 (1.15, 6.27)	1.68 (0.87, 2.24)	0.072	0.734
CA19−9 (U/mL)		8.71 (4.93, 24.78)	7.84 (4.66, 20.25)	0.574	15.08 (6.92, 24.54)	14.10 (6.48, 17.34)	0.795	0.183
AFP (ng/mL)		2.26 (1.55, 2.88)	2.08 (1.43, 2.44)	0.471	2.26 (1.74, 3.31)	2.21 (1.57, 3.01)	0.746	0.402
Tumor diameter (mm)		5.00 (3.88, 7.50)	7.00 (5.71, 8.50)	0.015^*^	5.00 (3.27, 7.00)	5.75 (3.23, 7.39)	0.570	0.078
Histological type^a^	Tubular Ade	34 (59.65%)	22 (75.86%)	0.293	18 (72.00%)	12 (100.00%)	0.173	0.191
	Poorly cohesive/signet ring cell Car	18 (31.58%)	4 (13.79%)		4 (16.00%)	0 (0.00%)		
	Mucinous Ade	3 (5.26%)	1 (3.45%)		2 (8.00%)	0 (0.00%)		
	Mixed Ade	2 (3.51%)	2 (6.90%)		0 (0.00%)	0 (0.00%)		
	Others	0 (0.00%)	0 (0.00%)		1 (4.00%)	0 (0.00%)		
Differentiation degree^b^	non-poorly differentiated	27 (47.37%)	13 (44.83%)	0.823	14 (56.00%)	9 (75.00%)	0.306	0.111
	poorly differentiated	30 (52.63%)	16 (55.17%)		11 (44.00%)	3 (25.00%)		
TNM stage^c^	I-II	11 (19.30%)	12 (41.38%)	0.029^*^	5 (20.00%)	7 (58.33%)	0.029^*^	0.521
	III-IV	46 (80.70%)	17 (58.62%)		20 (80.00%)	5 (41.67%)		
Perineural invasion	Negative	24 (42.11%)	25 (86.21%)	<0.001^*^	8 (32.00%)	10 (83.33%)	0.005^*^	0.395
	Positive	33 (57.89%)	4 (13.79%)		17 (68.00%)	2 (16.67%)		
Lymphovascular invasion	Negative	21 (36.84%)	11 (37.93%)	0.921	12 (48.00%)	6 (50.00%)	1.000	0.236
	Positive	36 (63.16%)	18 (62.07%)		13 (52.00%)	6 (50.00%)		

Quantitative data were presented as median (25th, 75th percentiles), using the Mann-Whitney U test. Categorical data were presented as n (%), using the chi-square test or Fisher exact test. AFP, Alpha-fetoprotein; Ade., Adenocarcinoma; CEA, Carcinoembryonic antigen; CA19−9, Carbohydrate antigen 19–9; CA125, Carbohydrate antigen 125; Car., Carcinoma; TNM, Tumor-lymph node-metastasis.

a Histological type was evaluated according to the fifth edition of the World Health Organization classification.

b non-poorly differentiated type included well/moderately differentiated and papillary adenocarcinomas, poorly differentiated type included poorly differentiated adenocarcinoma, poorly cohesive/signet-ring cell carcinoma, mucinous carcinoma, and others.

c TNM stage was evaluated according to the fifth edition of the World Health Organization classification.

^#^Comparison between MSI and MSS groups. ^†^Comparison between training and validation cohorts. ^*^P <0.05.

### Observer measurement consistency

The parameters of the lesions measured by the two observers were in good agreement (ICC > 0.75), as shown in **Table 2**. The measurement results of observer 1 was taken for further analysis.

**Table 2:** Consistency test of each parameter measured by two observers.

Parameter	MSI (*n*=41)		ICC	MSS (*n*=82)		ICC
	Observer 1	Observer 2		Observer 1	Observer 2	
ICAP (100µɡ/cm^3^)	15.98 ± 4.12	15.73 ± 4.21	0.956	17.68 (14.10,21.28)	17.55 (13.67,21.18)	0.938
nICAP	0.10 (0.09,0.12)	0.10 (0.08,0.12)	0.994	0.11 (0.09,0.14)	0.11 (0.09,0.13)	0.991
ICVP (100µɡ/cm^3^)	19.75 (18.14,23.69)	20.13 (18.62,22.99)	0.943	24.69 (20.87,29.39)	24.47 (21.42,29.22)	0.875
nICVP	0.42 ± 0.09	0.43 ± 0.11	0.919	0.48 (0.41,0.53)	0.48 (0.39,0.54)	0.816
ICEP (100µɡ/cm^3^)	19.28 ± 3.60	19.53 ± 3.89	0.972	22.16 (18.51,26.77)	22.08 (18.43,26.86)	0.889
nICEP	0.56 ± 0.09	0.57 ± 0.10	0.972	0.66 ± 0.15	0.66 ± 0.16	0.863
ECV (%)	35.29 ± 6.10	35.69 ± 6.26	0.971	41.95 ± 10.01	41.87 ± 9.61	0.873

Data are presented as median (25th, 75th percentiles) or mean ± standard deviation. AP, arterial phase; CI, confidence interval; EP, equilibrium phase; ECV, extracellular volume; IC, iodine concentration; ICC, intraclass correlation coefficient; MSI, microsatellite instability; MSS, microsatellite stability; nIC, normalized iodine concentration; VP, venous phase.

### Comparison of CT features and DECT-derived parameters

**Figure 2:**
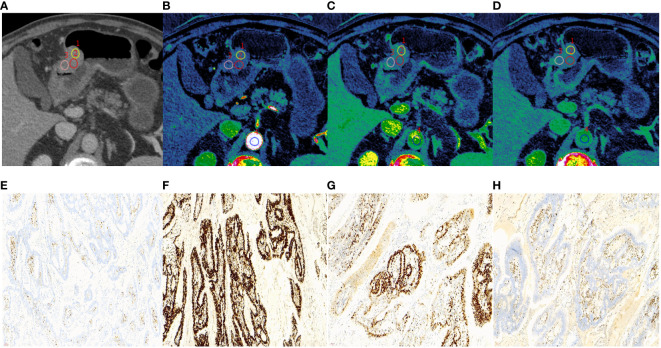
A 65-year-old male with histopathologically proved MSI gastric cancer. **(A)** 70keV monochromatic image in venous phase. A Bormann type I advanced gastric cancer was located in gastric antrum. **(B–D)** Iodine concentration pseudocolor images in arterial **(B)**, venous **(C)**, and equilibrium **(D)** phases. IC values were 11.53 100µɡ/cm^3^, 19.02 100µɡ/cm^3^, and 18.91 100µɡ/cm^3^, respectively. The corresponding nIC values were 0.07, 0.28, and 0.51, respectively. The ECV fraction after calculation was 31.06%. **(E–H)** Immunohistochemical results showed MLH1-negative **(E)**, MSH2-positive **(F)**, MSH6-positive **(G)**, PMS2-negative **(H)** (×200 magnification). MSI, microsatellite instability; IC, iodine concentration; nIC, normalized iodine concentration; ECV, extracellular volume.

**Figure 3:**
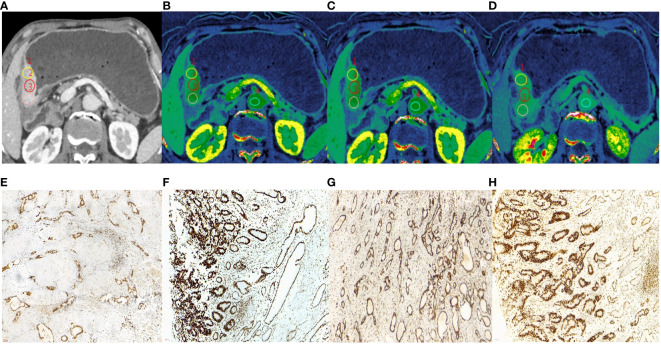
A 62-year-old male with histopathologically proved MSS gastric cancer. **(A)** 70keV monochromatic image in venous phase. A Bormann type IV advanced gastric cancer was located in gastric antrum. **(B–D)** Iodine concentration pseudocolor images in arterial **(B)**, venous **(C)**, and equilibrium **(D)** phases. IC values were 16.72 100µɡ/cm^3^, 25.62 100µɡ/cm^3^, and 26.56 100µɡ/cm^3^, respectively. The corresponding nIC values were 0.09, 0.49, and 0.71, respectively. The ECV fraction after calculation was 42.18%. **(E–H)** Immunohistochemical results showed MLH1-positive **(E)**, MSH2-positive **(F)** MSH6-positive **(G)**, PMS2-positive **(H)** (×200 magnification). MSS, microsatellite stability; IC, iodine concentration; nIC, normalized iodine concentration; ECV, extracellular volume.

In both training and validation cohorts, the ECV fraction, ICVP, ICEP, and nICEP of the MSI group were lower than those of the MSS group, and Borrmann type I-II were found more often in the MSI group (all *P* < 0.05) (**Figures 2**, **3**). In the training cohort, the incidence of gastric antrum cancer in the MSI group was higher, and the ICAP and nICVP in the MSI group were lower than those in the MSS group (all *P* < 0.05). Other variables showed no statistically significant differences in both cohorts (all *P >*0.05) (**Table 3**).

**Table 3:** Comparison of CT parameters between MSI and MSS groups in the training and validation cohorts.

Parameter		Training cohort (*n*=86)				Validation cohort (*n*=37)			
		MSI (*n*=29)	MSS (*n*=57)	*t/Z/χ^2^*	*P*	MSI (*n*=12)	MSS (*n*=25)	*t/Z/χ^2^*	*P*
Tumor thickness (cm)		1.70 (1.40, 2.00)	1.60 (1.20, 1.83)	-1.069	0.285	1.60 (1.20, 1.87)	1.60 (1.17, 1.90)	-0.292	0.770
Tumor location	Cardia/fundus	0 (0.00%)	9 (15.79%)	-2.042	0.041^*^	0 (0.00%)	2 (8.00%)	-0.438	0.661
	Body	7 (24.14%)	18 (31.58%)			3 (25.00%)	6 (24.00%)		
	Antrum/pylorus	22 (75.86%)	30 (52.63%)			9 (75.00%)	17 (68.00%)		
Borrmann type	I	7 (24.14%)	1 (1.75%)	3.161	0.002^*^	0 (0.00%)	1 (4.00%)	2.531	0.011^*^
	II	17 (58.62%)	30 (52.63%)			12 (100.00%)	10 (40.00%)		
	III	5 (17.24%)	20 (35.09%)			0 (0.00%)	13 (52.00%)		
	IV	0 (0.00%)	6 (10.53%)			0 (0.00%)	1 (4.00%)		
Serosal invasion	Negative	10 (34.48%)	11 (19.30%)	2.401	0.121	6 (50.00%)	5 (20.00%)	0.250	0.122
	Positive	19 (65.52%)	46 (80.70%)			6 (50.00%)	20 (80.00%)		
Lymph node metastasis	Negative	8 (27.59%)	10 (17.54%)	1.171	0.279	3 (25.00%)	3 (12.00%)	0.409	0.367
	Positive	21 (72.41%)	47 (82.46%)			9 (75.00%)	22 (88.00%)		
ICAP (100µɡ/cm^3^)		14.23 (12.02, 17.72)	18.08 (14.08, 21.31)	2.201	0.028^*^	16.73 (15.14, 17.89)	16.36 (14.74, 21.42)	0.081	0.935
nICAP		0.10 (0.08, 0.12)	0.11 (0.09, 0.13)	0.681	0.496	0.10 (0.08, 0.11)	0.12 (0.09, 0.15)	1.265	0.206
ICVP (100µɡ/cm^3^)		19.73 (17.98, 23.67)	24.67 (19.63, 29.50)	2.206	0.027^*^	19.90 (18.94, 23.70)	24.71 (22.89, 28.87)	2.920	0.004^*^
nICVP		0.41 (0.36, 0.48)	0.49 (0.41, 0.53)	2.453	0.014^b*^	0.44 (0.37, 0.48)	0.46 (0.41, 0.57)	1.363	0.173
ICEP (100µɡ/cm^3^)		18.92 (16.35, 21.77)	22.29 (17.36, 26.51)	2.37	0.018^*^	19.16 (18.31, 20.28)	21.65 (19.05, 27.43)	2.498	0.012^*^
nICEP		0.56 (0.51, 0.61)	0.66 (0.54, 0.74)	2.973	0.003^*^	0.55 ± 0.11	0.67 ± 0.12	2.962	0.005^*^
ECV (%)		34.86 (31.41, 38.45)	41.17 (36.34, 46.32)	3.056	0.002^*^	35.25 ± 7.83	43.84 ± 9.04	2.817	0.008^*^

Normally distributed quantitative data were presented as mean ± standard deviation, using the independent t test. Skewed distributed quantitative data were presented as median (25th, 75th percentiles), using the Mann-Whitney U test. Categorical data were presented as n (%), using the chi-square test or Fisher exact test. AP, arterial phase; EP, equilibrium phase; ECV, extracellular volume; IC, iodine concentration; MSI, microsatellite instability; MSS, microsatellite stability; nIC, normalized iodine concentration; VP, venous phase.

^*^P <0.05.

### Nomogram construction and performance evaluation

**Figure 4:**
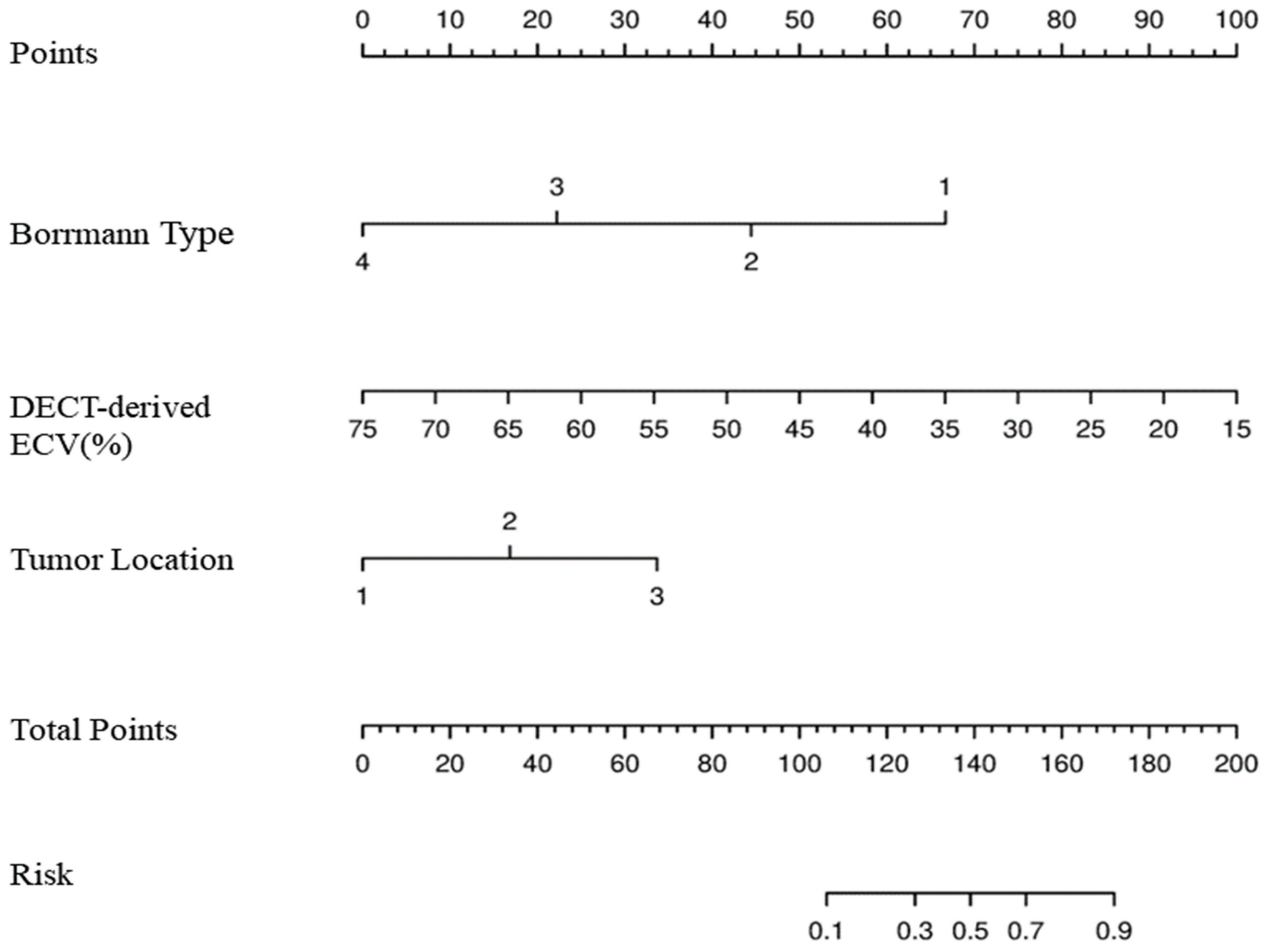
Development of nomogram for predicting MSI status. The nomogram was constructed in the training cohort via Borrmann type, DECT-derived ECV, and tumor location. In Borrmann type, the increasing numbers indicate I, II, III, and IV, respectively. In tumor location, 1 is cardia/fundus, 2 is gastric body, 3 is antrum/pylorus. MSI, microsatellite instability; DECT, dual-energy CT; ECV, extracellular volume.

**Figure 5:**
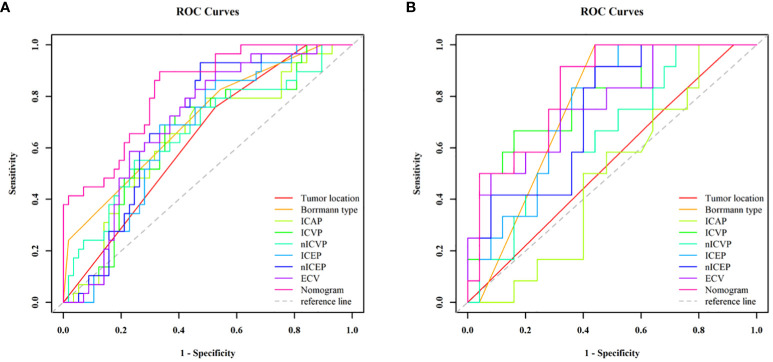
ROC curve analyses of each individual variable and nomogram to predict MSI status in the training cohort **(A)** and validation cohort **(B)**. The nomogram showed the highest AUC value of 0.826 (95% CI, 0.729–0.899) in the training cohort and 0.833 (95% CI, 0.675–0.933) in the validation cohort. MSI, microsatellite instability; ROC, receiver operating characteristic; AUC, areas under the curve.

**Figure 6:**
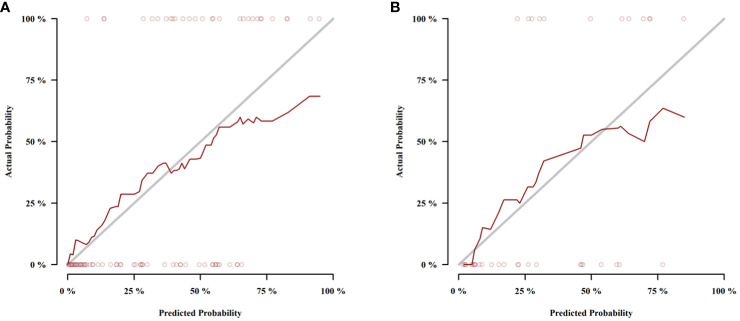
Calibration curves of the nomogram in the training cohort **(A)** and validation cohort **(B)**.The dotted lines (representing the nomogram) were close to the solid lines (representing an ideal model) in both cohorts, indicating the nomogram was well fitted.

**Figure 7:**
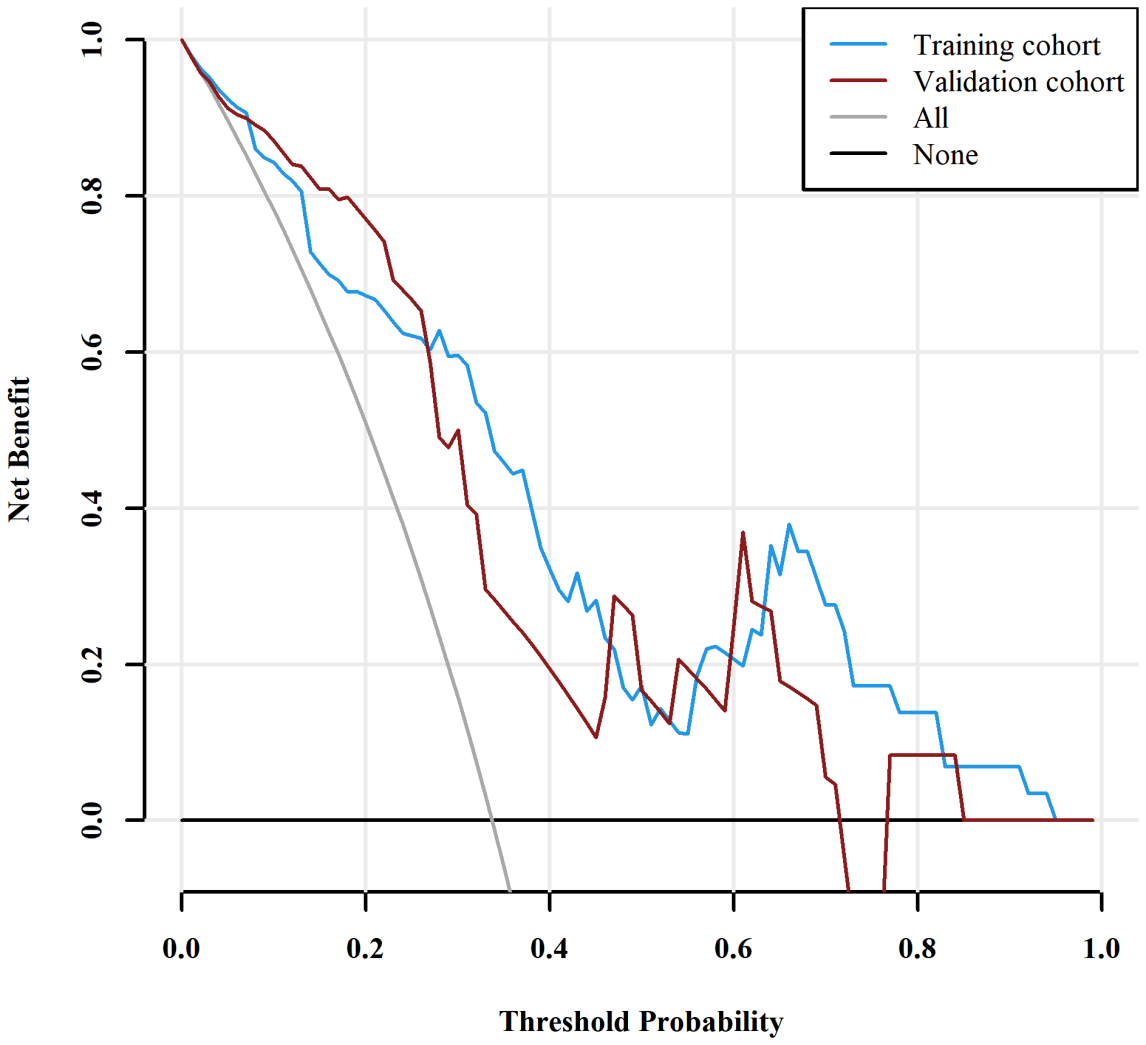
Decision curve analysis (DCA) for the nomogram. The DCA indicated that the nomogram achieved more net benefits within the most of thresholds probabilities than either the treat-all scheme (assuming all lesions are MSI) or the treat-none scheme (assuming all lesions are MSS). MSI, microsatellite instability; MSS, microsatellite stability.

Those variables which were shown to be statistically different by univariate analysis in the training cohort (tumor location, Borrmann type, ECV fraction, ICAP, ICVP, nICVP, ICDP, and nICDP) were included in multivariable logistic regression analysis, and tumor location, Borrmann type, and ECV fraction were identified as independent predictors of MSI (**Table 4**). A prediction model was subsequently developed based on the above independent predictors and visualized as a nomogram (**Figure 4**). In the training cohort, the nomogram yielded an AUC of 0.826 (95% CI, 0.729–0.899), significantly higher than the AUCs of all significant single variables; in the validation cohort, the nomogram yielded an AUC of 0.833 (95% CI, 0.675–0.935), significantly higher than the AUCs of tumor location, ICAP, and nICVP (all P < 0.05) (**Tables 5**, **6**; **Figure 5**). There was no significant difference of the nomogram between the AUCs in the two cohorts (P=0.925), suggesting no overfitting or underfitting of the nomogram. The calibration curves illustrated high consistency between the nomogram-predicted and actual observed probabilities (**Figure 6**), and the Hosmer-Lemeshow tests showed no statistically significant differences in the training and validation cohorts (P = 0.146 and 0.849, respectively). The DCA demonstrated that the nomogram obtained higher net benefits than the treat-none and treat-all strategies across most of the range of threshold probabilities (**Figure 7**).

**Table 4:** Multivariate logistic regression analysis for MSI status in GC in the training cohort.

Variables	β	OR	95% CI	P
intercept	3.8710			
Tumor location	1.1251	3.078	1.057–8.964	0.039
Borrmann type	-1.4848	0.226	0.085–0.601	0.003
ECV	-0.1113	0.895	0.828–0.967	0.005

β is the regression coefficient. CI, confidence interval; ECV, extracellular volume; OR, odds ratio.

**Table 5:** Diagnostic efficiencies of DECT-based variables and the nomogram.

Variables	Training cohort				Validation cohort			
	AUC (95% CI)	Cut-off value	Sensitivity (%)	Specificity (%)	AUC (95% CI)	Cut-off value	Sensitivity (%)	Specificity (%)
Tumor location	0.635(0.524–0.736)	2.00	75.86	47.37	0.545(0.373–0.709)	1.00	100.00	8.00
Borrmann type	0.709(0.601–0.802)	2.00	82.76	45.61	0.760(0.592–0.885)	2.00	100.00	56.00
ICAP (100 µɡ/cm^3^)	0.646(0.535–0.746)	17.60	75.86	54.39	0.508(0.339–0.676)	17.94	83.33	40.00
ICVP (100 µɡ/cm^3^)	0.646(0.536–0.746)	22.89	75.86	59.65	0.800(0.636–0.913)	22.18	66.67	84.00
nICVP	0.662(0.552–0.761)	0.41	55.17	75.44	0.640(0.466–0.791)	0.54	100.00	28.00
ICEP (100 µɡ/cm^3^)	0.657(0.547–0.756)	19.87	68.97	66.67	0.757(0.588–0.882)	22.93	100.00	48.00
nICEP	0.697(0.588–0.791)	0.65	93.10	52.63	0.740(0.570–0.870)	0.64	91.67	56.00
ECV (%)	0.702(0.594–0.796)	39.14	79.31	57.89	0.770(0.603–0.892)	38.45	75.00	68.00
Nomogram	0.826(0.729–0.899)	-0.94	89.66	66.67	0.833(0.675–0.935)	1.16	91.67	68.00

AUC, area under the curve; AP, arterial phase; CI, confidence interval; EP, equilibrium phase; ECV, extracellular volume; IC, iodine concentration; nIC, normalized iodine concentration; VP, venous phase.

**Table 6:** Comparison of AUCs between nomogram and individual variables in the training and validation cohorts.

Nomogram		Tumor location	Borrmann type	ICAP	ICVP	nICVP	ICEP	nICEP	ECV
Training cohort	*Z*	3.576	2.796	2.741	2.689	2.294	2.848	2.280	2.258
	*P*	<0.001^*^	0.005^*^	0.006^*^	0.007^*^	0.022^*^	0.004^*^	0.023^*^	0.024^*^
Validation cohort	*Z*	3.587	1.369	3.252	0.332	2.454	0.766	1.419	1.112
	*P*	<0.001^*^	0.171	0.001^*^	0.740	0.014^*^	0.444	0.156	0.266

AP, arterial phase; EP, equilibrium phase; ECV, extracellular volume; IC, iodine concentration; nIC, normalized iodine concentration; VP, venous phase.

^*^P <0.05.

## Discussion

In this study, we developed and validated a nomogram for MSI prediction in GC patients based on three independent predictors: tumor location, Borrmann type, and DECT-derived ECV fraction. The nomogram showed good discerning ability with AUCs of 0.826 in the training and 0.833 in validation cohorts, higher than that of significant single variables. The nomogram has the potential to be used as a noninvasive and easy-to-use tool for risk stratification, personalized management, and prognosis improvement in the GC population.

The ECV fraction, comprising the intravascular space and extravascular extracellular volume fractions (19), can provide valuable information for the changes in tumor tissue composition. Nishimuta et al. (23) found that the tumor ECV fraction determined by contrast-enhanced CT is closely associated with the tumor infiltration pattern of GC. However, the ECV fraction measurement using contrast-enhanced CT requires both pre- and post-contrast CT imaging, which would increase radiation dose and the risk of image misregistration (14). In contrast, ECV measurement using DECT only requires the iodine concentrations within the target tissue and aorta during the equilibrium phase, reducing the radiation dose to the patient as well as alignment errors due to positional changes before and after conventional CT enhancement. The virtual non-contrast imaging technology of DECT is a strong support for the clinical realization of this advantage. Additionally, ICs can more effectively and reliably quantify iodine contrast distribution in tissues than CT values (24). Previous studies (14, 25, 26) indicated that DECT-derived ECV showed better diagnostic performance than conventional CT-derived ECV.

Our study showed that the DECT-derived ECV fraction of the MSI group was lower than that of the MSS group, and the ECV fraction was an independent predictor of MSI. Three possible causes may account for this phenomenon. First, numerous studies have confirmed that the ECV fraction is strongly correlated with the amount of desmoplastic stroma (15, 16). Meanwhile, it is well known that GC is characterized by an abundant desmoplastic stroma reaction. Cancer-associated fibroblasts (CAFs) can secrete and remodel the fibrous stroma to promote the invasion and metastasis of GC (27). Compared to MSS GC, MSI GC has lower expression levels of CAF-related genes and less fibrous stroma deposition (28, 29), which may result in the reduction of extracellular volume and the decrease of ECV fraction. Furthermore, the incidences of earlier TNM stage and negative perineural invasion in the MSI group were higher than those in the MSS group in training and validation cohorts, which are consistent with previous findings (3, 6), indicating the less aggressiveness of MSI GCs. The lower level of angiogenesis in low-aggressive GCs, as evidenced by the lower IC values in the MSI group in this study, may contribute to the decreased ECV fraction, because it can decrease the size of intravascular space and blood leakage into the interstitial space. The third possible cause is the microstructure differences in tumor tissues. In line with previous studies (30, 31), the MSI group was composed of more tubular adenocarcinomas and fewer poorly cohesive carcinomas than the MSS group, although the difference did not reach statistical significance. Meanwhile, the hypermutated phenotype of MSI GC can stimulate the recruitment and activation of more tumor-infiltrating lymphocytes (TILs) (32). The tighter arrangement of tumor cells and higher density of TILs increase the complexity of the microstructure of diseased tissues and further restrict the expansion of extracellular space, resulting in lower ECV fraction for MSI groups than MSS groups. Similar to our study, most results demonstrated tumors with a higher aggressiveness had a higher ECV fraction (17, 18). However, not all studies have supported the above theory. For instance, Fukukura et al. (19) found that increased tumor ECV fractions were associated with better prognoses in patients with pancreatic adenocarcinoma. The discrepancy in these results may be related to structural differences between different tumors. Different from GC, pancreatic adenocarcinoma is regarded as a hypovascular tumor, which may affect the accumulation of contrast agents in extracellular space.

ICs can quantify tumor vascularization by reflecting the content and distribution of iodine contrast in tissues (33). Univariate analysis showed that IC parameters derived from fast kV DECT platform were lower in the MSI group than those in the MSS group, which is consistent with Zhu et al’s results obtained using dual-layer spectral-detector CT (DLCT) (34), indicating that IC parameters can be used to assess MSI status and is less affected by different DECT systems, which might be related to the fact that MSI GC downregulates the expression of angiogenesis-related genes and inhibits tumor angiogenesis (35, 36). However, different from the results of Zhu et al. (34), multivariable logistic regression showed that no IC parameter was identified as an independent predictor in our study, indicating that the differences in blood supply between different types of GC are smaller than the differences in extracellular space. The possible reasons for this result are discussed below. First, compared with IC parameters, the ECV is affected not only by blood flow but also by the extracellular matrix, which can comprehensively reflect the peculiar inflammatory tumor microenvironment of MSI GC. Second, the ICs in tumors are related not only to their own blood flow, blood volume and microvasculature but also to the scanning techniques (contrast medium and CT scanning protocols) and physiological variations (such as patient body weight and hemodynamic status). However, ECV fraction was regarded as a more robust quantitative parameter because it is insensitive to imaging acquisition parameters, contrast material injection dose and rate, systemic bias, and physiological variants (14, 37, 38).

Apart from the quantitative data, we investigated the value of CT features in MSI status assessment. As a result, tumor location and Borrmann type were identified as independent predictors and incorporated into the nomogram. The current study suggested that MSI GCs occurred predominantly in the antrum, which has been well verified by previous studies (39, 40). This might be related to different oncogenic inheritance pathways for GC at various locations (5). Borrmann types reflect the aggressiveness of GC by depicting tumor morphological features in the gastric mucosa and infiltration scale within the gastric wall. Borrmann type I and II GC have an earlier TNM stage and a better prognosis (41). The findings from previous clinicopathologic studies have indicated that type I and II were more likely to be seen in MSI GCs (42, 43); our results further validated the association. The earlier TNM stage and negative perineural invasion of the MSI group in this study suggested its less aggressiveness, which may be the main reasons for differences in tumor morphological features between the two groups.

Chen et al. (8) constructed a nomogram incorporating gender, age, tumor size, and normalized tumor enhancement ratio derived from venous-enhanced CT to evaluate MSI in GC and yield moderate predictive performance. However, their nomogram lacked a quantitative indicator and independent validation. Zhu et al. (34) proposed a nomogram based on DLCT in arterial and venous phases, which showed promising potential in predicting MSI in GC. Their nomogram included tumor location, CT-N staging, and DLCT model. However, their study neglected the contribution of the equilibrium phase scan. Compared with the DLCT model generated based on the regression equation of multiple parameters, the ECV fraction in our nomogram is easier to operate and more stable. Additionally, our study indicated that the ECV fraction may be superior to IC parameters in the description of the microenvironment in GC.

The present study has some limitations. First, this was a retrospective single-center study with a small sample size. A larger sample size and multicenter dataset are needed to validate the generalization capability of the proposed nomogram. Second, the optimal delay timing for measuring ECV fraction has yet to be well defined. Taking into account the circulation variations between individuals, we used the bolus tracking technique to determine the timing of contrast-enhanced CT scans. The equilibrium phase scans were acquired at 90s after the portal venous phase scanning ended, approximately 180–190s after contrast injection, which is in accordance with the timing for evaluating ECV in previous studies of rectal (17, 44) and pancreatic cancer (19, 45). Although prolonging the timing of equilibrium phase scans may be beneficial for even distribution of contrast agent between intravascular and extravascular–extracellular spaces, too long a delay scan time can interfere with routine clinical work. Yoon et al. (46) reported that an equilibrium phase 180s after contrast administration to estimate ECV is a good compromise between clinical workflow and technical success. Therefore, we accepted the existing scan protocol based on this argument. Thirdly, the hematocrit values in some cases were not obtained on the same day as the CT examinations, which may have an influence on the ECV fraction measurements. Finally, although ECV fraction was regarded as a robust indicator, it can still be disturbed by confounding factors, such as edema from inflammation, venous congestion, and tumor necrosis. Although we carefully avoided areas of tumor necrosis, invisible micronecrosis may have affected our results. Further research is needed to confirm the usefulness of ECV in large populations.

In summary, this study provides valuable insight into the potential value of DECT-derived ECV fraction in predicting MSI status. Furthermore, the proposed nomogram incorporating ECV fraction and CT features achieved excellent prediction ability and may be used in routine clinical practice as a noninvasive and easy-to-use indicator to identify MSI GCs. This might be conducive to reducing the time and cost brought by PCR and IHC, and assisting clinicians in making clinical decisions for patients with GC.

## Data availability statement

The original contributions presented in the study are included in the article/supplementary material. Further inquiries can be directed to the corresponding author.

## Ethics statement

The studies involving humans were approved by the Medical Ethics Committee of The First Affiliated Hospital of Dalian Medical University. The studies were conducted in accordance with the local legislation and institutional requirements. The ethics committee/institutional review board waived the requirement of written informed consent for participation from the participants or the participants’ legal guardians/next of kin because the national legislation and the institutional requirements.

## Author contributions

WH: Writing – review & editing, Writing – original draft, Validation, Software, Methodology, Investigation, Formal Analysis, Conceptualization. YZ: Writing – review & editing, Software, Resources, Methodology. HJ: Writing – review & editing, Visualization, Investigation. AC: Writing – review & editing, Methodology, Conceptualization. QX: Writing – review & editing, Visualization, Methodology. YL: Writing – review & editing, Resources, Data curation. ZZ: Writing – review & editing, Visualization, Investigation. AL: Writing – review & editing, Visualization, Supervision, Project administration, Investigation, Conceptualization.

## References

[1] Sung H Ferlay J Siegel RL Laversanne M Soerjomataram I Jemal A. Global cancer statistics 2020: globocan estimates of incidence and mortality worldwide for 36 cancers in 185 countries. CA Cancer J Clin. (2021) 71:209–49. doi: 10.3322/caac.21660 33538338

[2] Chen W Zheng R Baade PD Zhang S Zeng H Bray F. Cancer statistics in China, 2015. CA Cancer J Clin. (2016) 66:115–32. doi: 10.3322/caac.21338 26808342

[3] Ratti M Lampis A Hahne JC Passalacqua R Valeri N. Microsatellite instability in gastric cancer: molecular bases, clinical perspectives, and new treatment approaches. Cell Mol Life Sci. (2018) 75:4151–62. doi: 10.1007/s00018-018-2906-9 PMC6182336 30173350

[4] Arora S Balasubramaniam S Zhang W Zhang L Sridhara R Spillman D. Fda approval summary: pembrolizumab plus lenvatinib for endometrial carcinoma, a collaborative international review under project orbis. Clin Cancer Res. (2020) 26:5062–7. doi: 10.1158/1078-0432.Ccr-19-3979 32295834

[5] Liang X Wu Y Liu Y Yu D Huang C Li Z. A multicenter study on the preoperative prediction of gastric cancer microsatellite instability status based on computed tomography radiomics. Abdominal Radiol (New York). (2022) 47:2036–45. doi: 10.1007/s00261-022-03507-3 35391567

[6] Puliga E Corso S Pietrantonio F Giordano S. Microsatellite instability in gastric cancer: between lights and shadows. Cancer Treat Rev. (2021) 95:102175. doi: 10.1016/j.ctrv.2021.102175 33721595

[7] Cao Q Lai SY Xu N Lu Y Chen S Zhang XS. Computed tomography features of gastric cancer patients with DNA mismatch repair deficiency. Front Oncol. (2021) 11:619439. doi: 10.3389/fonc.2021.619439 33816249 PMC8012908

[8] Chen JY Tong YH Chen HY Yang YB Deng XY. Shao GL. A Noninvasive Nomogram Model Based Ct Features to Predict DNA Mismatch Repair Deficiency Gastric Cancer. Front Oncol. (2023) 13:1066352. doi: 10.3389/fonc.2023.1066352 36969034 PMC10034198

[9] Chung HW Lee SY Han HS Park HS Yang JH Lee HH. Gastric cancers with microsatellite instability exhibit high fluorodeoxyglucose uptake on positron emission tomography. Gastric Cancer. (2013) 16:185–92. doi: 10.1007/s10120-012-0165-2 22692466

[10] Zhao H Gao J Bai B Wang R Yu J Lu H. Development and external validation of a non-invasive imaging biomarker to estimate the microsatellite instability status of gastric cancer and its prognostic value: the combination of clinical and quantitative ct-imaging features. Eur J Radiol. (2023) 162:110719. doi: 10.1016/j.ejrad.2023.110719 36764010

[11] Zeng Q Zhu Y Li L Feng Z Shu X Wu A. Ct-based radiomic nomogram for preoperative prediction of DNA mismatch repair deficiency in gastric cancer. Front Oncol. (2022) 12:883109. doi: 10.3389/fonc.2022.883109 36185292 PMC9523515

[12] Jiang Z Xie W Zhou X Pan W Jiang S Zhang X. A virtual biopsy study of microsatellite instability in gastric cancer based on deep learning radiomics. Insights into Imaging. (2023) 14:104. doi: 10.1186/s13244-023-01438-1 37286810 PMC10247640

[13] Forghani R Srinivasan A Forghani B. Advanced tissue characterization and texture analysis using dual-energy computed tomography: horizons and emerging applications. Neuroimaging Clin N Am. (2017) 27:533–46. doi: 10.1016/j.nic.2017.04.007 28711211

[14] Fukukura Y Kumagae Y Higashi R Hakamada H Nakajo M Maemura K. Extracellular volume fraction determined by equilibrium contrast-enhanced dual-energy ct as a prognostic factor in patients with stage iv pancreatic ductal adenocarcinoma. Eur Radiol. (2020) 30:1679–89. doi: 10.1007/s00330-019-06517-w 31728691

[15] Bandula S Punwani S Rosenberg WM Jalan R Hall AR Dhillon A. Equilibrium contrast-enhanced ct imaging to evaluate hepatic fibrosis: initial validation by comparison with histopathologic sampling. Radiology. (2015) 275:136–43. doi: 10.1148/radiol.14141435 25490188

[16] Treibel TA Bandula S Fontana M White SK Gilbertson JA Herrey AS. Extracellular volume quantification by dynamic equilibrium cardiac computed tomography in cardiac amyloidosis. J Cardiovasc Comput Tomogr. (2015) 9:585–92. doi: 10.1016/j.jcct.2015.07.001 PMC4684159 26209459

[17] Luo Y Liu L Liu D Shen H Wang X Fan C. Extracellular volume fraction determined by equilibrium contrast-enhanced ct for the prediction of the pathological complete response to neoadjuvant chemoradiotherapy for locally advanced rectal cancer. Eur Radiol. (2023) 33:4042–51. doi: 10.1007/s00330-022-09307-z 36462046

[18] Adams LC Jurmeister P Ralla B Bressem KK Fahlenkamp UL Engel G. Assessment of the extracellular volume fraction for the grading of clear cell renal cell carcinoma: first results and histopathological findings. Eur Radiol. (2019) 29:5832–43. doi: 10.1007/s00330-019-06087-x 30887194

[19] Fukukura Y Kumagae Y Higashi R Hakamada H Takumi K Maemura K. Extracellular volume fraction determined by equilibrium contrast-enhanced multidetector computed tomography as a prognostic factor in unresectable pancreatic adenocarcinoma treated with chemotherapy. Eur Radiol. (2019) 29:353–61. doi: 10.1007/s00330-018-5570-4 29922930

[20] Li J Fang M Wang R Dong D Tian J Liang P. Diagnostic accuracy of dual-energy ct-based nomograms to predict lymph node metastasis in gastric cancer. Eur Radiol. (2018) 28:5241–9. doi: 10.1007/s00330-018-5483-2 29869176

[21] Kim HJ Kim AY Oh ST Kim JS Kim KW Kim PN. Gastric cancer staging at multi-detector row ct gastrography: comparison of transverse and volumetric ct scanning. Radiology. (2005) 236:879–85. doi: 10.1148/radiol.2363041101 16020558

[22] Pan Z Pang L Ding B Yan C Zhang H Du L. Gastric cancer staging with dual energy spectral ct imaging. PloS One. (2013) 8:e53651. doi: 10.1371/journal.pone.0053651 23424614 PMC3570537

[23] Nishimuta Y Tsurumaru D Kai S Maehara J Asayama Y Oki E. Extracellular volume fraction determined by equilibrium contrast-enhanced computed tomography: correlation with histopathological findings in gastric cancer. Japanese J Radiol. (2023) 41:752–9. doi: 10.1007/s11604-023-01393-3 PMC10313564 36735208

[24] Chen Y Shi K Li Z Wang H Liu N Zhan P. Survival prediction of hepatocellular carcinoma by measuring the extracellular volume fraction with single-phase contrast-enhanced dual-energy ct imaging. Front Oncol. (2023) 13:1199426. doi: 10.3389/fonc.2023.1199426 37538109 PMC10394647

[25] Zhou Y Geng D Su GY Chen XB Si Y Shen MP. Extracellular volume fraction derived from dual-layer spectral detector computed tomography for diagnosing cervical lymph nodes metastasis in patients with papillary thyroid cancer: A preliminary study. Front Oncol. (2022) 12:851244. doi: 10.3389/fonc.2022.851244 35756662 PMC9213667

[26] Ohta Y Kitao S Watanabe T Mukai N Kishimoto J Yamamoto K. Measurement of myocardial extracellular volume fraction from iodine density images using single-source, dual-energy computed tomography: A feasibility study. J Comput Assist Tomogr. (2017) 41:750–6. doi: 10.1097/rct.0000000000000587 28240638

[27] Ham IH Lee D Hur H. Role of cancer-associated fibroblast in gastric cancer progression and resistance to treatments. J Oncol. (2019) 2019:6270784. doi: 10.1155/2019/6270784 31281359 PMC6590541

[28] Mak TK Li X Huang H Wu K Huang Z He Y. The cancer-associated fibroblast-related signature predicts prognosis and indicates immune microenvironment infiltration in gastric cancer. Front Immunol. (2022) 13:951214. doi: 10.3389/fimmu.2022.951214 35967313 PMC9372353

[29] Zhang C Sun D Li C Liu Y Zhou Y Zhang J. Development of cancer-associated fibroblasts subtype and prognostic model in gastric cancer and the landscape of tumor microenvironment. Int J Biochem Cell Biol. (2022) 152:106309. doi: 10.1016/j.biocel.2022.106309 36174922

[30] Arai T Sakurai U Sawabe M Honma N Aida J Ushio Y. Frequent microsatellite instability in papillary and solid-type, poorly differentiated adenocarcinomas of the stomach. Gastric Cancer. (2013) 16:505–12. doi: 10.1007/s10120-012-0226-6 23274922

[31] Mathiak M Warneke VS Behrens HM Haag J Böger C Krüger S. Clinicopathologic characteristics of microsatellite instable gastric carcinomas revisited: urgent need for standardization. Appl Immunohistochem Mol Morphol. (2017) 25:12–24. doi: 10.1097/pai.0000000000000264 26371427 PMC5147042

[32] Kim KJ Lee KS Cho HJ Kim YH Yang HK Kim WH. Prognostic implications of tumor-infiltrating foxp3+ Regulatory T cells and cd8+ Cytotoxic T cells in microsatellite-unstable gastric cancers. Hum Pathol. (2014) 45:285–93. doi: 10.1016/j.humpath.2013.09.004 24331841

[33] Chen XH Ren K Liang P Chai YR Chen KS Gao JB. Spectral computed tomography in advanced gastric cancer: can iodine concentration non-invasively assess angiogenesis? World J Gastroenterol. (2017) 23:1666–75. doi: 10.3748/wjg.v23.i9.1666 PMC5340819 28321168

[34] Zhu Y Wang P Wang B Jiang Z Li Y Jiang J. Dual-layer spectral-detector ct for predicting microsatellite instability status and prognosis in locally advanced gastric cancer. Insights into Imaging. (2023) 14:151. doi: 10.1186/s13244-023-01490-x 37726599 PMC10509117

[35] Miyamoto N Yamamoto H Taniguchi H Miyamoto C Oki M Adachi Y. Differential expression of angiogenesis-related genes in human gastric cancers with and those without high-frequency microsatellite instability. Cancer Lett. (2007) 254:42–53. doi: 10.1016/j.canlet.2007.02.004 17374440

[36] Qing X Xu W Liu S Chen Z Ye C Zhang Y. Molecular characteristics, clinical significance, and cancer immune interactions of angiogenesis-associated genes in gastric cancer. Front Immunol. (2022) 13:843077. doi: 10.3389/fimmu.2022.843077 35273618 PMC8901990

[37] Chang CC Lin CY Chu CY Hsiung YC Chuang MT Tseng YL. Extracellular volume fraction measurement correlates with lymphocyte abundance in thymic epithelial tumors. Cancer Imaging. (2020) 20:71. doi: 10.1186/s40644-020-00349-4 33028413 PMC7539449

[38] Kim PK Hong YJ Sakuma H Chawla A Park JK Park CH. Myocardial extracellular volume fraction and change in hematocrit level: mr evaluation by using T1 mapping in an experimental model of anemia. Radiology. (2018) 288:93–8. doi: 10.1148/radiol.2018171342 29613847

[39] Cristescu R Lee J Nebozhyn M Kim KM Ting JC Wong SS. Molecular analysis of gastric cancer identifies subtypes associated with distinct clinical outcomes. Nat Med. (2015) 21:449–56. doi: 10.1038/nm.3850 25894828

[40] Choi YY Kim H Shin SJ Kim HY Lee J Yang HK. Microsatellite instability and programmed cell death-ligand 1 expression in stage ii/iii gastric cancer: *post hoc* analysis of the classic randomized controlled study. Ann Surg. (2019) 270:309–16. doi: 10.1097/sla.0000000000002803 29727332

[41] Díaz Del Arco C Ortega Medina L Estrada Muñoz L Molina Roldán E Cerón Nieto M García Gómez de Las Heras S. Are borrmann's types of advanced gastric cancer distinct clinicopathological and molecular entities? A western study. Cancers (Basel). (2021) 13:3081. doi: 10.3390/cancers13123081 34205546 PMC8234739

[42] Miceli R An J Di Bartolomeo M Morano F Kim ST Park SH. Prognostic impact of microsatellite instability in asian gastric cancer patients enrolled in the artist trial. Oncology. (2019) 97:38–43. doi: 10.1159/000499628 31048579

[43] Lee HS Choi SI Lee HK Kim HS Yang HK Kang GH. Distinct clinical features and outcomes of gastric cancers with microsatellite instability. Mod Pathol. (2002) 15:632–40. doi: 10.1038/modpathol.3880578 12065777

[44] Sun Q Bian X Sun D Wang M Dong H Dai X. The value of preoperative diagnosis of colorectal adenocarcinoma pathological T staging based on dual-layer spectral-detector computed tomography extracellular volume fraction: A preliminary study. Japanese J Radiol. (2024). doi: 10.1007/s11604-024-01537-z 38381249

[45] Fukui H Onishi H Nakamoto A Tsuboyama T Ota T Yano K. Pancreatic fibrosis by extracellular volume fraction using contrast-enhanced computed tomography and relationship with pancreatic cancer. Eur J Radiol. (2022) 156:110522. doi: 10.1016/j.ejrad.2022.110522 36113381

[46] Yoon JH Lee JM Klotz E Jeon JH Lee KB Han JK. Estimation of hepatic extracellular volume fraction using multiphasic liver computed tomography for hepatic fibrosis grading. Invest Radiol. (2015) 50:290–6. doi: 10.1097/rli.0000000000000123 25493416

